# The Pharmacology of Pain Associated With the Monoiodoacetate Model of Osteoarthritis

**DOI:** 10.3389/fphar.2019.00974

**Published:** 2019-09-18

**Authors:** João de Sousa Valente

**Affiliations:** Vascular Biology and Inflammation Section, Cardiovascular School of Medicine and Sciences, British Heart Foundation Centre of Excellence, King’s College London, London, United Kingdom

**Keywords:** monoiodoacetate, osteoarthritis pain, animal models of pain, pharmacology of osteoarthritis, cartilage

## Abstract

The high incidence of osteoarthritis (OA) in an increasingly elderly population anticipates a dramatic rise in the number of people suffering from this disease in the near future. Because pain is the main reason patients seek medical help, effective pain management—which is currently lacking—is paramount to improve the quality of life that OA sufferers desperately seek. Good animal models are, in this day and age, fundamental tools for basic research of new therapeutic pathways. Several animal models of OA have been characterized, but none of them reproduces entirely all symptoms of the disease. Choosing between different animal models depends largely on which aspect of OA one aims to study. Here, we review the current understanding of the monoiodoacetate (MIA) model of OA. MIA injection in the knee joint leads to the progressive disruption of cartilage, which, in turn, is associated with the development of pain-like behavior. There are several reasons why the MIA model of OA seems to be the most adequate to study the pharmacological effect of new drugs in pain associated with OA. First, the pathological changes induced by MIA share many common traits with those observed in human OA (Van Der Kraan et al., 1989; Guingamp et al., 1997; Guzman et al., 2003), including loss of cartilage and alterations in the subchondral bone. The model has been extensively utilized in basic research, which means that the time course of pain-related behaviors and histopathological changes, as well as pharmacological profile, namely of commonly used pain-reducing drugs, is now moderately understood. Also, the severity of the progression of pathological changes can be controlled by grading the concentration of MIA administered. Further, in contrast with other OA models, MIA offers a rapid induction of pain-related phenotypes, with the cost-saving consequence in new drug screening. This model, therefore, may be more predictive of clinical efficacy of novel pharmacological tools than other chronic or acute OA models.

## Introduction

The *Textbook of Rheumatology* defines osteoarthritis (OA) as a “slowly progressive monoarticular [ … ] disorder of unknown cause and obscure pathogenesis” affecting primarily the hands and weight-bearing joints such as hips and knees (Firestein et al., 2016). It is defined clinically by joint pain, deformity, and loss of function and pathologically by articular cartilage loss and remodeling of the subchondral bone. With the advent of better imaging techniques, synovitis is being increasingly recognized as being present in a considerable proportion of cases (Sokolove and Lepus, 2013; Xie et al., 2019). OA is the most common form of arthritis or degenerative joint disease; affecting millions of people (Bijlsma et al., 2011), with the World Health Organization estimating that, globally, up to 10% of people over the age of 60 years is affected by some form of OA (Hunter et al., 2014). There is currently no cure for the disease, with currently available treatment focusing on temporary symptomatic pain relief and alleviating inflammation, often leaving patients with considerable pain and functional disability. Paracetamol, non-steroidal anti-inflammatory drugs (NSAIDs), and steroids are the most prescribed pain therapies (Lee et al., 2004). Patients that do not respond to NSAIDs are candidates for opioid therapy. These therapeutic options come, however, with severe side effects: prolonged NSAID use can lead to gastrointestinal bleeding and renal toxicity and increase cardiovascular risks, and opioids are associated with constipation and potential for addiction (Maniar et al., 2018). For patients with end-stage OA, surgical joint replacement is required (Hunter and Felson, 2006). Pain management in OA continues to be one of the main focuses of research because pain is the main reason why OA patients seek medical care. However, there is currently no drug that can fully treat OA-related pain; a better understanding of the pathophysiological mechanisms in play in OA is crucial if we are to deliver better treatment options to these patients.

## Animal Models of OA Pain: Surgical and Chemical Models

To study OA in the laboratory setting, several animal models have been developed over the last decades that contributed to a better understanding of the pathological mechanisms behind the disease. There are obvious limitations with these models, particularly those related to differences in anatomy, gait, and cartilage characteristics compared to human joints. The models only mimic parts or stages of the disease, with no model completely reproducing human OA complexity. Despite this, the use of animal models allows the study of the disease within controlled environment parameters and tissue collection at different time points of the model (Lampropoulou-Adamidou et al., 2014). We can divide OA animal models into two large groups—spontaneous models and induced models. Spontaneous models develop slowly but are pathophysiologically closest to human OA. However, due to the spontaneous nature of these models, it is challenging to find appropriate age-matched controls for pharmacological studies. Further, they are time- and money-consuming to produce and have a high maintenance cost.

In the second group—induced models—there are chemically and/or surgically induced animal models of OA. Surgical models include damage to the anterior cruciate ligament and partial or complete menisectomy. These models have been validated in many species and consist of induced lesions similar to those observed in humans; therefore, it is expected that the disease will progress in a similar way to the progression of post-traumatic human OA. Some more aggressive surgical models are, however, not appropriate to study the effect of drugs targeting pain, because of the rapid progression of cartilage degeneration and slow and inconsistent development of pain-related behavior. Additionally, surgical models are often technically challenging. Nevertheless, surgical models have been used with great success, as seen in recent studies that looked at the pain mechanism in OA (Mapp et al., 2013; Nwosu et al., 2016; Ashraf et al., 2018).

Chemically induced OA models, on the other hand, require much less intervention, consisting, normally, of a single intra-articular injection of substances, such as monoiodoacetate (MIA), papain, or mucilage, that can target different components of the joint (Lampropoulou-Adamidou et al., 2014; Micheli et al., 2019). Because of their artificial onset, these models do not recapitulate the natural onset of the human disease but have, nevertheless, found pre-clinical value, namely because they originate robust and reproducible pain phenotypes, making them particularly adequate to test the efficacy of new pharmacological agents to treat OA pain (Bove et al., 2003; Micheli et al., 2019). In addition, chemically induced models are easy to implement and require less invasive procedures than surgically induced models. Further, because of the fast onset of development of the pain phenotype—which can be controlled by controlling the dosage of the substances injected—they are much less expensive than spontaneous models. Of all OA models, the MIA model is the most often used, being commonly chosen to study the efficacy of pharmacological agents to treat pain (Bove et al., 2003; Fernihough et al., 2004).

## The Monoiodoacetate Model

MIA intra-articular injection results in histopathological alterations and functional impairment similar to some of the features observed in the early phases of human OA. MIA is an inhibitor of glyceraldehyde-3-phosphate, disrupting cellular glycolysis, which in turn leads to eventual cell death (Sabri and Ochs, 1971; Van Der Kraan et al., 1989). Because the site of injection is restricted to the joint space, intra-articular injection of MIA causes mainly chondrocyte cell death, leading to cartilage degeneration and subsequent subchondral bone alterations (Guingamp et al., 1997; Janusz et al., 2001). Although MIA can potentially affect different types of cells in the joint, the avascular nature of cartilage makes chondrocytes particularly vulnerable (Dunham et al., 1992; Guzman et al., 2003). While the method of induction is not technically challenging, MIA is highly toxic if it enters the circulation, quickly resulting in the animal’s death, so care should be taken not to pierce the joint capsule during the injection, so as to prevent MIA from leaking outside of the joint.

## Pathophysiology of MIA

### Joint

**Figure 1:**
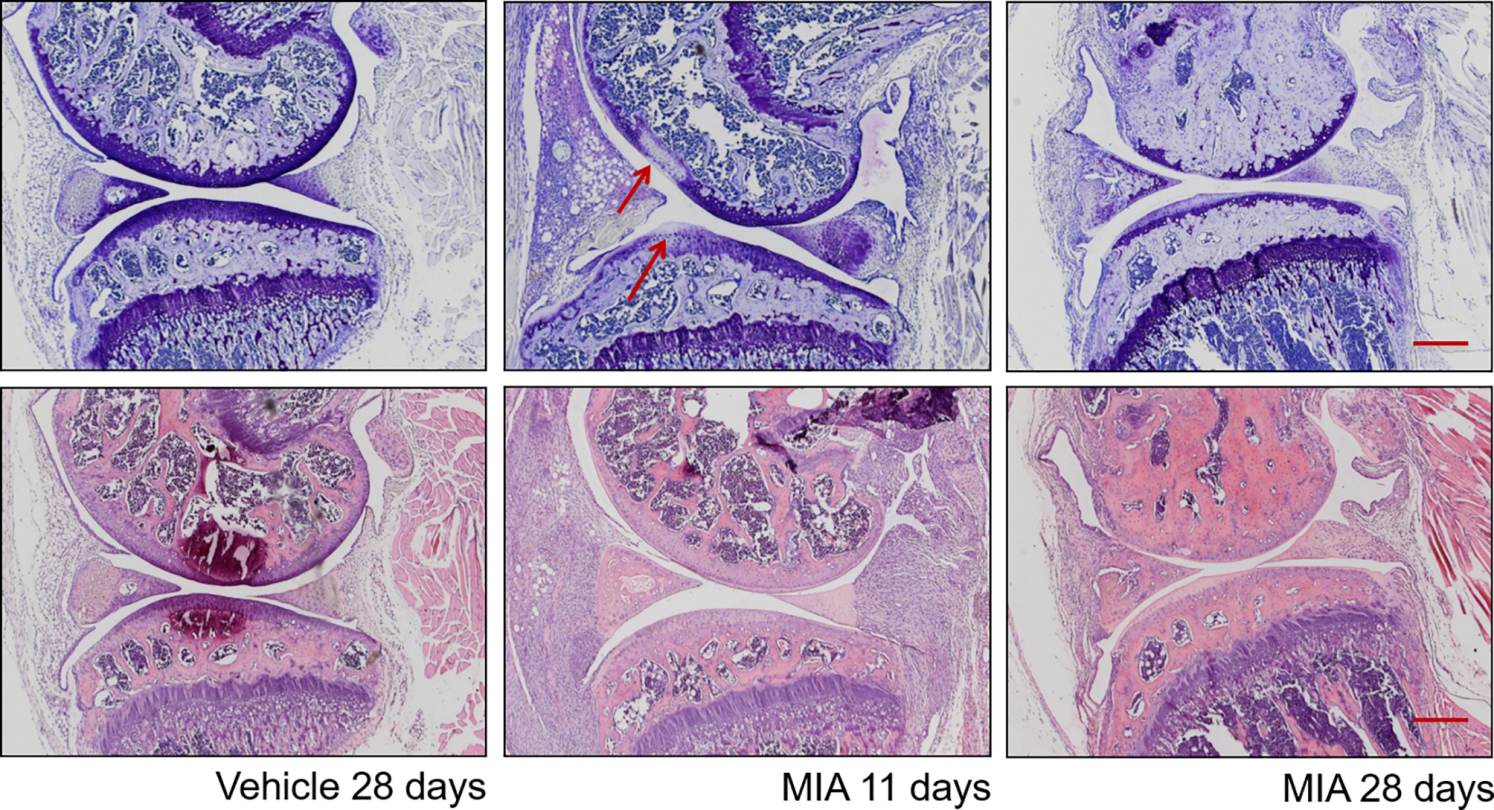
Histopathological progression in the monosodium iodoacetate model of osteoarthritis. Top plane: Representative sections at different time points post–MIA injection (1 mg/mouse) stained with toluidine blue/purple, with femoral condyle at the top and tibial plateau at the bottom. (Left) Vehicle-injected joint with full-depth normal cartilage and normal subchondral bone structure. (Middle) Eleven days after MIA injection, visible focal cartilage damage and loss of proteoglycan staining (arrows) in both femoral and tibial condyles. (Right) Twenty-eight days after MIA injection, marked thinning of the whole articular surface, loss of proteoglycan staining, and restructuring of subchondral bone. Bottom plane: Representative sections at different time points post–MIA injection (1 mg/mouse) stained with hematoxylin and eosin. (Left) Vehicle-injected joint with normal synovium and few inflammatory cells visible. (Middle) At 11 days, there are obvious signs of inflammation: the synovial membrane is expanded, with a significantly increased density of inflammatory cells. (Right) At 28 days, inflammation is reduced, with a significant decrease in synovial size, but a dense cellularity is still observable. Scale bar = 100 µm.

Structurally, as early as 1 day after MIA injection, alterations to the surrounding synovium and articular cartilage have been described (**Figure 1**) (Bove et al., 2003; Guzman et al., 2003; Orita et al., 2011). Chondrocytes are shrunken with fragmented nuclei, and some areas of chondrocyte degeneration are present 1 to 3 days post-injection. The early stage of the model’s progression is characterized by signs of inflammation such as synovial membrane expansion and infiltration of macrophages, neutrophils, mast cells, lymphocytes, and plasma cells, but they normally subside at day 7 (**Figure 1**) (Sousa-Valente et al., 2018). In accordance, human OA is also associated with synovitis, characterized by increased infiltration of macrophages and other immune cell types (Hill et al., 2007; Scanzello and Goldring, 2012) as well as elevation of cytokine levels in synovial OA samples (Smith et al., 1997). Proximal structures such as the meniscus and ligaments also show signs of inflammation. In accordance, after MIA injection, an elevation of pro-inflammatory mediators such as Tumor Necrosis Factor-α (TNF-α) and Interleukin 6 (IL-6) is observed, typically peaking at day 4 (Orita et al., 2011). Clinically used NSAIDs given locally can reduce MIA-induced pain as well as MIA-induced C- and A-fiber spontaneous activity (Schuelert and Mcdougall, 2009; Kelly et al., 2012). Later stages of the disease (after day 10–14) are characterized by progressive cartilage degradation and remodeling of subchondral bone. Fourteen days post-injection, areas of full-thickness cartilage damage have been characterized. Formation of osteoclasts has also been described (Guzman et al., 2003). There is also evidence of osteochondral angiogenesis and vascularization (Walsh et al., 2010; Ashraf et al., 2011). Recently, it was also shown that superoxide dismutase mimetic compound MnIIMe2DO2A can reduce pain sensitivity and TNF-alpha serum levels in an MIA ankle model, suggesting a role of oxygen reactive species in the late stage of the model (Di Cesare Mannelli et al., 2013).

### Primary Sensory Afferents

The knee joint is innervated by primary sensory neurons (PSNs). Because cartilage is aneural, mechanisms independent of cartilage loss participate in mediating the initial pain in this model. However, in later stages, sensory nerves have been described to grow into the cartilage, along with new blood vessels (Ashraf et al., 2011). Nerve fibers detecting bone marrow lesions and edema have been shown to contribute to OA pain (Schaible and Grubb, 1993). PSN afferents can become sensitized by agents such as histamine or cytokines, underlying spontaneous pain, hyperalgesia, and allodynia following intra-articular MIA injection (Woolf and Ma, 2007). In accordance, MIA induces a concentration-dependent increase of afferent responses to mechanical stimulation (Schuelert and Mcdougall, 2009). Joint cells such as synoviocytes, inflammatory cells, or chondrocytes produce chemokines, cytokines, and proteases, which can sensitize PSN afferents (Schaible et al., 2009). Li et al. have demonstrated that when dorsal root ganglia neurons are co-cultured with synovial fluid from OA patients, there is a clear elevation of genes associated with neuronal pathways (e.g., Substance P (SP), Neurokinin (NK1), Neurokinin (NK2), Neuropeptide y receptor (NPYR1), Neuropeptide y receptor (NPYR2), α2δ1) or inflammation Cyclooxygenase 2 (COX2)/Prostaglandin-endoperoxide synthase 2 (PTGS2) and IL-6/interferon β2), suggesting that blocking inflammation can be a way of modulating OA pain (Li et al., 2011). Knee joints of both human and rodents are highly innervated by peptidergic afferents, i.e., they contain the peptides substance P and/or calcitonin gene-related peptide (CGRP) (Saito and Koshino, 2000; Sousa-Valente and Brain, 2018), and the number of CGRP-positive fibers in the joint is increased in the OA joint (Sousa-Valente et al., 2018), a feature also observed in human hip OA (Saxler et al., 2007). Consistently, there is an increase of CGRP content in the cell bodies of PSNs innervating the joint as well as an increase of CGRP release from the central terminals of PSNs (Fernihough et al., 2005; Ferreira-Gomes et al., 2010; Sousa-Valente et al., 2018). Further, peripheral blockade of CGRP receptors by inhibitor BIBN4096BS alleviates MIA-induced weight-bearing deficits (Hirsch et al., 2013). Nerve growth factor (NGF), which is an important trophic factor, is increased in OA joints (Iannone et al., 2002; Manni et al., 2003), and pre-treatment with anti-NGF antibodies prevented the development of mechanical hypersensitivity in MIA-treated mice (Xu et al., 2016; Sousa-Valente et al., 2018). Indeed, anti-NGF therapies have shown promising analgesic potential for OA pain treatment, with NGF antibodies showing efficacy for pain relief (Lane et al., 2010). Interestingly, NGF released from cells in the joint can increase CGRP and SP expression in Tropomyosin receptor kinase A (TrkA)-expressing neurons (Malcangio et al., 1997), potentially linking the roles of CGRP and NGF in OA pain (Malcangio et al., 1997; Ogbonna et al., 2013; Sousa-Valente et al., 2018).

### Spinal Cord

Primary afferent fibers of the knee joint project to several spinal cord segments and terminate in both the superficial and deeper laminae, where they synapse with dorsal horn neurons (Woolf and Ma, 2007). Pathological changes in the joint cause these dorsal horn neurons to become hyperexcitable (Neugebauer et al., 1993), reducing their thresholds and enhancing their responses to knee stimulation. Further, sensitized dorsal horn neurons expand their receptive fields, a mechanism that underlies the spread of hypersensitivity from the knee joint to adjacent areas. In accordance, MIA facilitates the responses of wide-dynamic-range (WDR) neurons to noxious and non-noxious stimulation (Chu et al., 2011). Further, MIA-induced increase in dorsal horn Fos immunoreactivity—a marker of neuronal activation—at both 7 and 28 days post–MIA injection correlates with behavioral outcomes (Sousa-Valente et al., 2018). MIA-induced pain is also associated with increased phosphorylation of mitogen-activated protein kinases (MAPK) in the dorsal horn of the spinal cord, and MAPK1 inhibitor PD98059 blocked both MIA-induced pain behavior and phosphorilated extracellular signal-regulated kinases 1/2 (pERK1/2) induction in the spinal cord (Lee et al., 2011). The transient receptor potential vanilloid type 1 ion channel (TRPV1) is a polymodal transducer receptor expressed on a subset of PSNs that responds to various stimuli such as noxious heat, protons, and molecules such as capsaicin. It plays a crucial role in the development of burning pain and reflex hyperactivity across several models of pathological pain, including OA pain, where TRPV1 expression is elevated (Fernihough et al., 2005; Nagy et al., 2014). Interestingly, TRPV1 activation modulates the firing of spinal nociceptive neurons in the MIA model, and blocking TRPV1 prevented spontaneous firing of WDR neurons (Chu et al., 2011). This mechanism involves the release of CGRP into the dorsal horn of the spinal cord (Puttfarcken et al., 2010), and consistently, intrathecal administration of CGRP antagonist CGRP8-37 ameliorates MIA-induced mechanical allodynia (Ogbonna et al., 2013). Another important player modulating the afferent input into the dorsal horn is the endocannabinoid system, with various components of the system being elevated in the spinal cord of MIA-treated animals and anandamide catabolism blockade using Fatty acid amide hydrolase (FAAH) inhibitor URB597 having inhibitory effects in MIA-induced mechanically evoked responses of WDR neurons (Sagar et al., 2010). Interestingly, endocannabinoid regulation of OA pain happens at multiple levels in the neuroaxis; in MIA-treated joints, local administration of Cannabinoid receptor 1 (CB1) receptor agonist Arachidonyl-2'-chloroethylamide (ACEA) reduces the mechanosensitivity of afferent nerve fibers. This effect is reduced by blocking either Cannabinoid receptor 1 (CB1) receptor or TRPV1, suggesting that both receptors crosstalk in cannabinoid-mediated antinociception (Schuelert and Mcdougall, 2008; Chen et al., 2016).

After MIA injection, a microglial response (microgliosis) in the ipsilateral dorsal horn, as well as microglia activation (p-p38 immunoreactivity), has been reported (Lee et al., 2011; Sagar et al., 2011; Sousa-Valente et al., 2018). Attenuation of microglial activation, *via* administration of glial inhibitor minocycline, is correlated with reduced pain behaviors in the MIA model (Sagar et al., 2011). In contrast with the established role of microglia activation and proliferation in the development of the MIA model of OA, the participation of astrocytosis is less clear, with some studies reporting a lack of astrocyte response (Lee et al., 2011; Ogbonna et al., 2013), while others studies report an increase of Glial fibrillary acidic protein (GFAP) immunoreactivity (Sagar et al., 2014; Lin et al., 2017).

## Neuropathic Component

OA patients commonly complain of referred pain, i.e., pain in areas adjacent to the affected joint (Bajaj et al., 2001; Khan et al., 2004) and in a subset of OA patients who continue to feel pain even after a technically successful joint replacements (Lundblad et al., 2008). Both circumstances suggest the existence of a neuropathic component to OA pain, given that the pain arises in areas outside the injury site or after the peripheral nociceptive input has been removed altogether. In animal models, there are also signs of a neuropathic component; the aforementioned microglial activation observed during the development of the MIA model is often observed in different models of peripheral nerve damage. Further, expression of the peripheral nerve damage marker AMP-dependent transcription factor (ATF-3) in the Dorsal root ganglion (DRG) has been described in the MIA model (Ferreira-Gomes et al., 2012; Thakur et al., 2012). Pharmacologically speaking, gabapentin has been shown to have an analgesic effect in the late phase of the model, when NSAIDs appear to lose efficacy (Fernihough et al., 2004; Ivanavicius et al., 2007).

## Behavior Profile of MIA

In addition to structural changes, MIA-induced pain-related behavior has been characterized. Pain assessment in animals is challenging. Commonly used assays such as the von Frey test or the dynamic plantar aesthesiometer are used to measure alterations of nociceptive mechanical thresholds in the hind paw, rather than the injected knee joint—a measurement of referred pain. This is, primarily, because measuring such thresholds from the joint is technically challenging. Nevertheless, as mentioned above, during experimental OA, joint afferents typically expand their receptive fields to areas adjacent to the injecting joint. The same expansion of receptive fields and reduction of mechanical thresholds around the joint area have been observed in human OA patients (Wessel, 1995; Farrell et al., 2000; Kosek and Ordeber, 2000). Another method commonly used is the incapacitance test, which measures the weight distribution between both hind limbs—a measurement of static pain (Bove et al., 2003). Weight-bearing asymmetry and paw withdrawal thresholds are measurements of ongoing pain and referred pain, respectively, and 1 mg MIA is the only dose to induce both, with 0.5 and 0.75 mg MIA only producing referred pain (Pomonis et al., 2005; Pitcher et al., 2016).

Interestingly, changes in the hind paw weight distribution closely followed changes in punctuate allodynia (Combe et al., 2004). MIA-induced pain-related behavior has a typical biphasic temporal profile (Pomonis et al., 2005; Pitcher et al., 2016). It usually manifests 1–3 days after administration as weight-bearing deficits and development of referred allodynia or hyperalgesia (Combe et al., 2004; Fernihough et al., 2004; Pomonis et al., 2005). This biphasic pattern is coincident with structural changes in the model progression, with the first stage associated with substantial inflammatory response and the second stage reflecting structural changes to the joint (Bove et al., 2003; Fernihough et al., 2004).

## Pharmacology of MIA

The biphasic nature of the model can be also observed in the responsiveness of the model to pharmacological agents (**Table 1**). The initial stage of the pain phenotype is sensitive to paracetamol (Fernihough et al., 2004) and NSAIDs, which seems to correlate with signs of inflammation (Guingamp et al., 1997; Bove et al., 2003; Fernihough et al., 2004; Pomonis et al., 2005). This stage is followed by a later phase that is NSAID-insensitive where morphine, tramadol, and gabapentin are more efficacious, suggesting a neuropathic component at this stage, as mentioned above (Combe et al., 2004; Fernihough et al., 2004; Pomonis et al., 2005; Ivanavicius et al., 2007). This profile is somewhat different from the one observed in human patients, where a continuous although partial efficacy of NSAIDs is observed. Due to the responsiveness of this animal OA model, extensive pharmacological profiling has been employed to unveil new therapeutic targets to treat OA (**Table 1**).

**Table 1:** Pharmacological modulation of pain-related behavior in MIA model of osteoarthritis.

Compound	Dose (mg/kg)	Observed changes in pain-related behavior		References
		Early phase	Late phase	
Diclofenac	30	M.H.	–	(Fernihough et al., 2004)
Morphine	6	M.A., M.H.	M.H., M.A., W.B.	(Combe et al., 2004; Fernihough et al., 2004; Pomonis et al., 2005)
Gabapentin	6	–	M.A., W.B	(Fernihough et al., 2004; Ivanavicius et al., 2007)
Paracetamol	1	M.H.	W.B.	(Fernihough et al., 2004) (Bove et al., 2003)
Naproxen	10	–	W.B.	(Bove et al., 2003)
Rofecoxib	10	–	W.B.	(Bove et al., 2003)
Tramadol	3	–	M.A., W.B.	(Combe et al., 2004)
CGRP8-37	5nmol/5 µl/mouse	–	M.A.	(Ogbonna et al., 2013)
Indomethacin	3	–	W.B.	(Pomonis et al., 2005)
Celecoxib	3	–	W.B.	(Pomonis et al., 2005)
A-796260*	35	–	M.E.	(Yao et al., 2008)
URB597**	5	–	W.B.	(Schuelert et al., 2011)
PF-04457845	0.3	–	M.H.	(Ahn et al., 2011)
Amitriptyline	3	–	W.B.	(Ivanavicius et al., 2007)
A-889425***	30	–	M.E.	(Chu et al., 2011)
Anti-NGF antibody	5	M.A.	–	(Sousa-Valente et al., 2018)
PD98059^+^	10 µl	–	M.E.	(Lee et al., 2011)
BIBN4096BS^++^	3	W.B.	–	(Hirsch et al., 2013)
Minocycline^+++^	30	–	M.A	(Sagar et al., 2011)

M.A, referred mechanical hyperalgesia; measured with von Frey apparatus; M.H, mechanical hyperalgesia, measured with Randall–Salito; W.B, weight bearing, measured with incapacitance tester; M.E, movement-evoked pain, grip force test.

*CB2 agonist.

**FAAH inhibitor.

***TRPV1 antagonist.

^+^MAPK1 inhibitor.

^++^CGRP antagonist.

^+++^glial cell inhibitor.

## Conclusion

OA causes movement impairment and progressive disability, therefore impacting severely the quality of life (Bijlsma et al., 2011; Chevalier et al., 2013). Given that aging and obesity are two of the main risk factors associated with OA, the prevalence is expected to increase by up to 50% in the future, highlighting the urgent need to find therapies to help manage OA (Hootman and Helmick, 2006; Perruccio et al., 2006; Hunter et al., 2014). The MIA model of OA is a well-established and widely used chemical model of OA, being characterized by a robust and rapid pain phenotype, which can be graded by altering the MIA dosage. Despite the lack of correlation of the model with the pathogenesis of human OA, there is similarity between the changes that occur as consequence of MIA injection and those observed in human OA, namely, development of synovitis, progressive cartilage degeneration, and subchondral bone alterations. Additionally, over the last decades, several studies helped us gain insight into different pathological processes in play—from changes in the joint *per se* to alterations in sensory afferents projecting to the joints and to plasticity changes at the spinal cord level—making this model interesting in unraveling the biological mechanisms underlying the development of OA pain. Furthermore, pharmacological interventions commonly used in the clinic to treat OA pain such as NSAIDs or paracetamol have been found to also improve MIA-induced pain, reinforcing the translational potential of this model as a model to test the clinical efficacy of novel pharmacological tools.

## Author Contributions

JV wrote the manuscript.

## Funding

This work was supported by Arthritis Research U.K. (ARUK21524).

## Conflict of Interest Statement

The author declares that the research was conducted in the absence of any commercial or financial relationships that could be construed as a potential conflict of interest.
